# A locally congenic backcross design in pig: a new regional fine QTL mapping approach miming congenic strains used in mouse

**DOI:** 10.1186/1471-2156-12-6

**Published:** 2011-01-14

**Authors:** Juliette Riquet, Hélène Gilbert, Bertrand Servin, Marie-Pierre Sanchez, Nathalie Iannuccelli, Yvon Billon, Jean-Pierre Bidanel, Denis Milan

**Affiliations:** 1 INRA, UMR 444 Laboratoire de Génétique Cellulaire, 31326 Castanet-Tolosan, France; 2 INRA, UMR1313 Génétique Animale et Biologie Intégrative, 78350 Jouy-en-Josas, France; 3 INRA, UE967 Génétique Expérimentale en Productions Animales, 17700 Surgères, France

## Abstract

**Background:**

In previous studies, a major QTL affecting fatness and growth has been mapped to pig chromosome 1q (SSC1q) using Large White - Meishan intercrosses. A higher fat depth and a larger growth rate have been reported for the allele of MS origin. Additionally the LW allele showed partial dominance effects over the MS allele for both traits. In order to refine the QTL mapping interval, advanced backcross generations were produced. Recombinant heterozygous sires were mated to LW sows in order to progeny test the sire segregation of the QTL and refine the QTL localisation. However due to the partial dominance of the LW allele, BC scheme using LW as the receiving population was not optimal.

**Results:**

To overcome the difficulties related to the dominance of the LW QTL allele, a population of dams locally homozygous for the MS haplotype in the QTL region, but with an overall 29/32 LW genetic background, has been set up. Progeny testing results, using these receiver dams, were much more significant than those previously obtained with LW dams, and the SSC1 QTL interval was refined to 8 cM. Considering the results obtained, a powerful experimental design for farm animals is proposed, mimicking locally genetically identical strains used in mouse for QTL fine mapping.

**Conclusions:**

We have further characterized the fatness QTL on pig chromosome 1 and refined its map position from a 30 cM interval to a 8 cM interval, using a locally congenic BC design. We have obtained highly significant results and overcome difficulties due to the dominance of the LW allele. This design will be used to produce additional, advanced BC families to further refine this QTL localization.

## Background

The development of genetic maps in livestock species over the last twenty years has spurred efforts to dissect the molecular basis of the genetic variation of economically important traits (QTL). Numerous chromosomal regions predicted to harbour genes influencing traits of interest in livestock populations have been identified using mapping experiments in outbred populations or in experimental crosses. However, in most cases, mapping resolution has been rather poor due to the limited number of markers, the limited size of experiments and the genetic heterogeneity of the investigated populations. In the majority of livestock populations, genetically homogenous lines do not exist. Nevertheless, recombinant progeny testing (RPT) has successfully been used in pigs for the fine mapping of QTL detected in experimental crosses between domestic European populations and either Wild Boar or Meishan animals [1,2]. It consists in backcrossing individuals carrying a distinguishable recombinant haplotype in the initial confidence interval of the QTL to individuals from one of the parental breeds for progeny testing. The location of the QTL is then determined relative to the recombinant points and the results of the segregation analyses in different families. At each generation, the haplotypes are traced through marker-assisted backcrossing and young boars with a smaller "exotic" segment in the initial QTL interval are retained to be progeny tested. Using this strategy and after 7 generations of backcrosses, the critical region for FAT1 QTL has been reduced from a 70 cM interval to a 3.3 cM interval on pig chromosome 4 [3]. Usually, the dams used are from the European parental breed because backcrosses using exotic breed (Wild Boar or Meishan) are economically difficult to sustain due to low growth rate and poor carcass quality of the animals.

However this strategy is based on two important assumptions. First, the two breeds are assumed to be each fixed for a different QTL allele (q or Q) associated with different phenotypic values. If this assumption does not hold and the introgressed allele from the exotic line (say q) is still segregating in the receiving dam line used to produce the backcross, a sire may have received the same allele from the two breeds. Based on the absence of QTL segregation in the progeny, the region defined by the exotic chromosomal segment of the sire will be then wrongly excluded from the QTL confidence interval.

Second, the power of RPT fine mapping design is largely influenced by the dominance relationship between QTL allele effects. In general, the introgressed q allele is supposed to be a (co)-dominant allele. The power of the RPT design is optimal when this assumption holds but is, on the contrary, null with a recessive allele. In practice, if the q allele is recessive with regard to the Q allele, no segregation can be observed when recombinant q/Q sires are back crossed to Q/Q dams, resulting in a false exclusion of the chromosomal segment harbouring the QTL. Partial dominance relationships would lead to mapping powers that are intermediate between these two extreme situations.

Among recently detected QTL, significant effects for growth, backfat depth, carcass, and meat quality traits have been identified in the telomeric region of the long arm of SSC1 in different F2 crosses between Meishan (MS) and Large-White (LW) breeds [4-9]. A higher fat depth and a larger growth rate have been reported for the allele of MS origin. Moreover, the LW allele showed partial dominance effects over the MS allele for growth and fatness traits. Up to now, the published confidence interval of this QTL is rather large (30 cM) and we propose here a BC fine mapping strategy, mimicking locally congenic strains in mice, which overcomes the difficulties due to the dominance of the LW QTL allele.

## Results and discussion

### Fine mapping of SSC1 QTL

**Figure 1:**
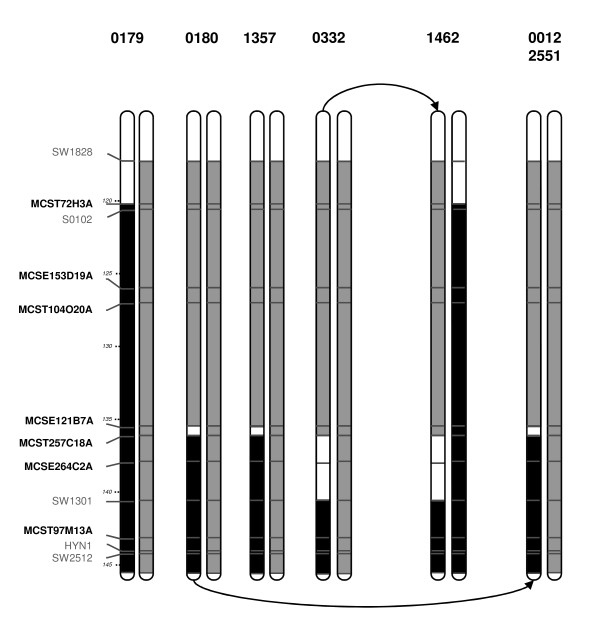
**Haplotypes of the different recombinant sires progeny tested**. Only the initial QTL interval between SW1828 and SW2512 microsatellites is reported on this figure. Position on the genetic map is indicated on the left. New microsatellites are indicated in black and bold. Haplotypes in QTL interval are represented for each sire, and breed origins are differentiated by colors (Meishan: black, Large White: grey, or unknown: white). All the MS segments have a common origin, LW chromosomes are unrelated. Recombinant chromosomes, identical by descent, are noted with an arrow.

Quantitative trait loci with strong effects on growth and body composition were detected in an F2 cross between Large White (LW) and Meishan (MS) pigs within the French PorQTL program [8,9]. Among the significant or suggestive effects found for different chromosomal regions, a QTL with large effects on backfat thickness was located on chromosome 1, with a rather large initial mapping interval (θ = 30 cM) delimited by SW1828 and SW2512 microsatellites. A QTL fine mapping program has been initiated at INRA in order to reduce this interval. An F1 sire from the initial F2 design, shown to be heterozygous for the QTL, was mated to LW sows to produce successive generations of backcross progeny. Three BC2 sires (#0179-#0180-#0332), found to be recombinant within the confidence interval of the QTL, were mated to LW dams in order to test the segregation of QTL alleles in their progeny (Figure 1). Significant likelihood ratio tests (LRT) were obtained for the BC2 sires at a 5% level for backfat thickness and meat percentage (Table 1). In addition these three families yielded significant likelihood ratio tests for fat depth G2 (#0180 and #0332 sires families at P < 1%) or lean depth M2 traits (family #0179 P < 5%). Under the assumptions that there is a single QTL affecting fatness and leanness and that these three BC2 sires were heterozygous, the QTL interval was thus reduced from MCST257C18A microsatellite to the end of SSC1 - (Figure 1).

**Table 1:** QTL detection results obtained for backfat thickness, meat percentage, G2 and M2 traits

Sire	Dams local genotype	**Trait**^**a**^	Number of offspring	Maximum LRT	Position	QTL Effect	Phenotypic SD	**Significance**^**b**^
**0179**	LW/LW	BFT	**147**	**8,9**	**140**	**-0,52**	**1,90**	*
		Meat percentage	78	9	130	0,69	1,63	*
		G2	78	5,9	132	-0,88	2,56	-
		M2	78	6,6	129	1,34	3,68	*
**0180**	LW/LW	BFT	**79**	**7,4**	**136**	**-0,69**	**1,97**	*
		Meat percentage	43	10,1	119	0,91	1,59	*
		G2	43	11,7	119	-1,49	2,39	**
		M2	43	0,5	136	-0,55	4,53	-
**0332**	LW/LW	BFT	**154**	**8,4**	**140,8**	**-0,50**	**1,92**	*
		Meat percentage	86	9,9	97,8	1,03	2,28	*
		G2	85	13,4	110,8	-1,04	1,90	**
		M2	85	5,5	100,8	1,06	3,44	-
**1357**	LW/LW	BFT	149	0,9	119	-0,16	1,85	-
		Meat percentage	140	0,8	128	-0,13	1,59	-
		G2	139	1,9	128	0,29	2,26	-
		M2	139	4	136	0,71	3,87	-

**1357**	MS/MS	BFT	**133**	**25,4**	**140**	**-1,09**	**2,19**	****
		Meat percentage	126	54,4	142	1,38	1,71	****
		G2	126	45	143	-1,95	2,73	****
		M2	126	16,1	151	1,43	3,52	***
**1462**	MS/MS	BFT	150	1,1	132	-0,22	2,25	-
		Meat percentage	121	4,2	134	0,37	1,70	-
		G2	121	3,2	134	-0,50	2,65	-
		M2	121	4,9	132	0,99	4,24	-
**2551**	MS/MS	BFT	**101**	**43,5**	**140**	**-1,63**	**2,08**	****
		Meat percentage	89	37,1	141	1,59	2,09	****
		G2	89	37,1	148	-1,98	2,51	****
		M2	89	9,1	148	1,23	3,44	*
**0012**	MS/MS	BFT	**177**	**49,8**	**138**	**-1,60**	**2,66**	****
		Meat percentage	115	23,6	140	1,28	2,51	****
		G2	115	31,6	141	-1,79	2,96	****
		M2	115	4,8	136	0,90	4,04	-

^a^: trait units are mm for G2, M2 et BFT (Back Fat Thickness) and % for Meat percentage.

^b^: - Non significant; * P < 0.05; ** P < 0.01, *** P < 0.003 and **** P < 5.10^-5^

Maximum likelihood ratio test value (Maximum LRT), corresponding significance level, position (cM), QTL substitution effects (in trait units), and within family standard deviation (SD) are given for the 7 sires retained in the scheme, depending on the dam genotypes in the chromosomic region of interest.

Additionally, a BC3 sire (#1357) was also progeny tested. Conversely, LRT were not significant for any trait when progeny testing was carried out using pure LW dams (Table 1). This result was not in accordance with the results obtained from the BC2 sires, BC3 sire (#1357) being heterozygous MS/LW in the previously defined interval. No likely localisation of the QTL could be deduced from combining the MS chromosomal portions of these four sires and their QTL estimated genotype.

**Figure 2:**
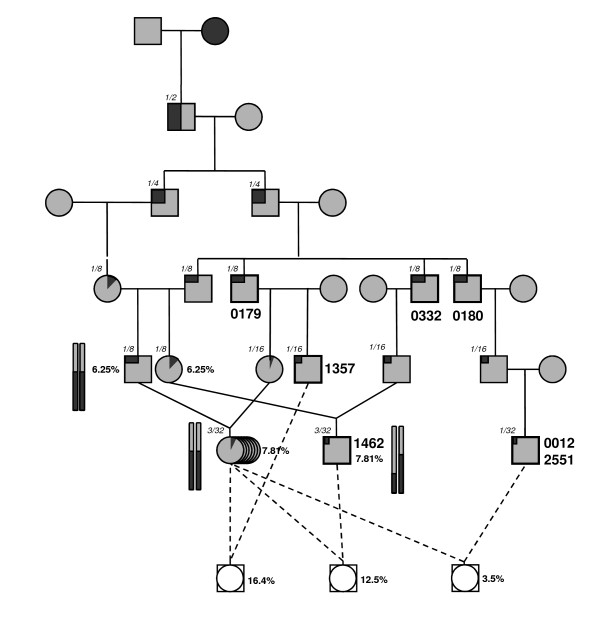
**Structure of the SSC1 pedigree**. The 7 different recombinant sires are noticed with a bold square and the sire name. Italic numbers indicate the MS proportion in the genome. The black chromosome pairs represent the individuals carrying a homozygote congenic MS chromosomal segment in the QTL region. The dotted lines indicated crosses realised between recombinant sires and locally MS/MS dams. For inbred animals, inbreeding coefficients is indicated.

The partial dominance of LW over MS alleles was a potential explanation for these inconsistent results. A much higher power would be obtained from backcrosses with MS dams, but matings with MS sows are complicated by early puberty and phenotype measurement is more expensive. In order to overcome these difficulties, a population of dams with a 29/32 LW genetic background, but locally homozygous in the QTL region for the MS haplotype carried by the above mentioned F1 boar, has been set up (Figure 2). The BC3 sire (#1357), which previously showed non significant results when mated with LW dams, was progeny tested again using these locally MS/MS dams. Highly significant results (P < 5.10^-5^) were obtained. Based on the progeny testing results obtained, the localisation interval of the QTL affecting fatness was defined by the only shared MS chromosomal fragment among the 4 BC sires that showed significant QTL effects. The QTL interval was decreased to approximately 8 cM, limited by MCST257C18A marker and the end of the chromosome. For all these sires the MS haplotype was associated with higher fat deposition, as expected.

The minimal chromosomal interval (136-144cM) obtained was later confirmed by progeny testing additional BC4 sires with locally MS/MS dams (Figure 1): as expected, no significant likelihood ratio tests values were obtained for a BC sire (#1462) which was homozygous MS/MS in the refined QTL region (Figure 1). Highly significant results (P < 0.005%) were obtained with two additional heterozygous BC4 sires (#0012 and #2551). For BFT trait, the LW/LW - MS/LW contrast amounted to 0.3 phenotypic standard deviation (SD) and the MS/MS - MS/LW contrast amounted to 0.7 SD on average, with an additive QTL effect of 0.5 SD, and a partial dominance effect (0.2 SD) of the LW allele (Table 1). Contrasts of 0.3 SD correspond to a maximum power of detection of 96% (with 154 progenies), whereas a power of 100% is reached for 0.7 SD with 80 progenies, confirming a risk of not detecting the QTL with LW/LW females. Note that for sire #1357 the estimated LW/LW - MS/LW contrast was 0.08 SD of the trait, corresponding to a power of detection reduced to 17% with its 149 progenies. The variability of the polygenic effects between sire families, as well as heterogeneity of the alleles carried out by the Large White females used for progeny testing, could explain the differences in significance levels between sire families.

### Theoretical scheme

**Figure 3:**
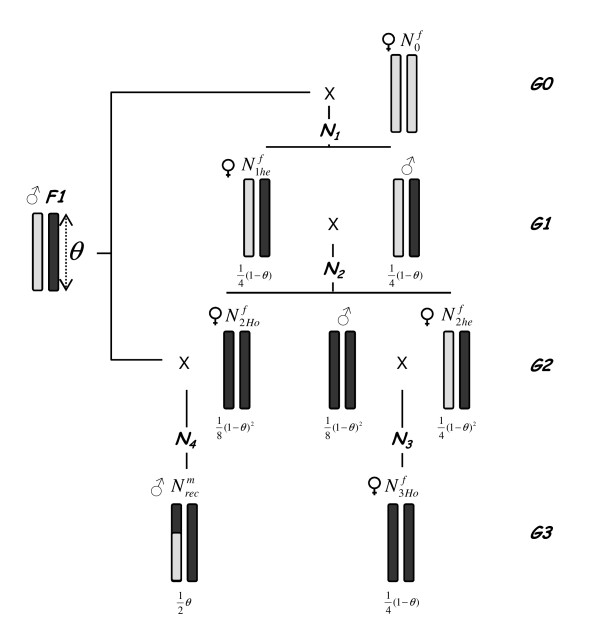
**Strategy to fine map a QTL located initially in *θ *cM segment S**. The black segments show Meishan origin, the grey segments Large White origin. All the MS segments have a common origin, LW chromosome are unrelated. The number of individuals in each sample $N_{*}^{*}$ (and the probability to obtain them depending on *θ *are indicated.

Based on this experience, we propose a mapping scheme, mimicking genetically identical strains used in mice for the fine mapping of QTL, to produce powerful experimental designs for farm animals. The objective is to set up, from a progeny tested F1 sire, a population dedicated to fine mapping, with a minimal haplotype variability in the region of interest along with a minimal inbreeding. It will be used for progeny testing sires that are recombinant in the initial QTL localisation interval. We propose to use a unique mating scheme to 1) produce dams that are locally homozygous for an identical by descent haplotype ("testor haplotype") in the chromosomal region of interest, 2) obtain recombinant sires that are carrying the same "testor haplotype" and a recombinant haplotype ("tested haplotype") for this region, and 3) mate the recombinant sires to the locally homozygous dams to predict the QTL genotype of the sire, by estimating the contrast between "testor" and "tested" haplotypes through a progeny test (Figure 3). In this example, a LW x Meishan F1 sire has been progeny tested and identified as heterozygous for the targeted QTL in a chromosomal region of size *θ *based on marker information. In the following, heterozygosity and homozygosity terms will only apply to this region. In G0 generation, the F1 sire is mated to LW ($N_{0}^{f}$) dams. Among their *N1 *offspring (G1 generation), heterozygous LW/MS sires are selected and mated to their heterozygous half-sisters ($N_{1he}^{f}$). The *N2 *progeny obtained in the G2 generation present all possible genotypes in the chromosomal region (homozygous MS/MS or LW/LW and heterozygous sires and dams). Among these *N2 *animals, (1) all locally homozygous LW piglets and heterozygous LW/MS males are discarded, (2) the ($N_{2Ho}^{f}$) homozygous dams are selected and mated to the founder F1 sire to produce G3 recombinant sires ($N_{rec}^{m}$) in the QTL mapping interval, and (3) heterozygous dams ($N_{2he}^{f}$) are mated with the locally homozygous G2 MS/MS sires to produce homozygous G3 MS/MS dams ($N_{3Ho}^{f}$). Those dams will be used to progeny test the ($N_{rec}^{m}$) sires.

Inbreeding is controlled if dams are produced using a rational Maximum Avoidance of Inbreeding (MAI) mating scheme [10]: *D *dam nuclei have to be constituted, so that sires from nucleus *d (d = 1,...,D) *are systematically mated with dams from nucleus *(d+g)*, *g *being the generation number. Following the proposed strategy, if *D *≥ 6, the relationship coefficients between G3 sires and dams will range from 0.18 to 0.23.

In the example proposed here, microsatellites markers were used to genotype all the individuals of the design. The interest of genetic markers in our scenario is to be informative to build the phases of the individuals of the pedigree and few multi-allelic markers are sufficient to obtain this information. Many biallelic markers instead of informative microsatellites would thus not change the power of the design, but at most help refining the location of the recombinant point of the progeny tested sires.

### Estimation of the various sample sizes

Each QTL is first positioned within few cM confidence interval (*θ*), with the QTL mapping design. Recombinant progeny testing is then used to decrease the chromosomic region containing QTL to a new resolution *δ*. Both the initial QTL mapping interval *θ *and the number of recombinant sires that are progeny tested ($N_{rec}^{m}$) determine the expected new resolution *δ*. The different samples sizes at each generation have been estimated from the number of recombinant sires ($N_{rec}^{m}$) required to significantly reduce the QTL confidence and simulations were computed for different $N_{rec}^{m}$ and *θ *values. Estimations were obtained for a typical pig type pedigree where 10 dams are used for the progeny test of each recombinant sire. The dependencies between the sample sizes at each step of the experiment were proven to be as in the following (Figure 3): $N_{rec}^{m}$ drives the number of dams $N_{2Ho}^{f}$ (homozygous MS females in generation G2 used as dams of the recombinant sires), and consequently ($N_{1he}^{f}$) (heterozygous females in generation G1) and $N_{0}^{f}$ (pure LW female in generation G0); and finally, through $N_{0}^{f}$, it determines $N_{2he}^{f}$ (heterozygous females used as dams to produced the homozygous MS females in generation G3) and the number of females available for the progeny tests, i.e. $N_{3Ho}^{f}$ (homozygous MS females mated with the recombinant sires for progeny testing).

**Figure 4:**
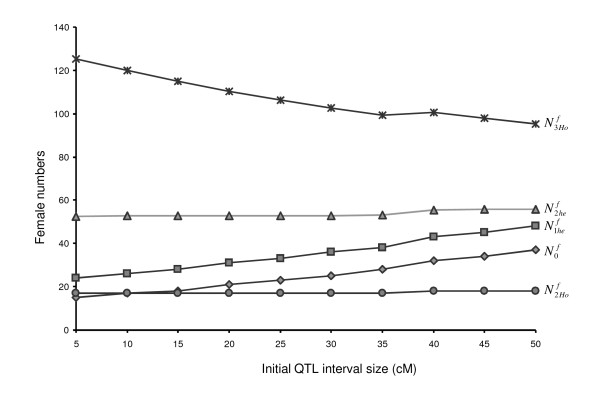
**Dam sample sizes as a function of the initial interval size *θ *to obtain a resolution *δ *of 2.5 cM**.

When considering different *θ *values in respect to a targeted resolution of 2.5 cM, the dam population sizes differed independently from the others (Figure 4). The number of females $N_{2Ho}^{f}$ necessary to produce the $N_{rec}^{m}$ sire was quite equivalent for the different *θ *values tested (from 17 to 18): for large *θ *values, a given $N_{rec}^{m}$ was easy to achieve; on the contrary, when *θ *values were small, the probability to obtain recombinant offspring was low, so that the number of litters required did not decrease. The number of females $N_{2he}^{f}$ did not vary much with the different *θ *values tested (from 53 to 56). Larger differences were observed for $N_{1he}^{f}$ and $N_{0}^{f}$ values for different *θ *values. When *θ *= 5 cM $N_{1he}^{f}$ and $N_{0}^{f}$ values were respectively 24 and 15, and increased to 48 ($N_{1he}^{f}$) and 37 ($N_{0}^{f}$) dams, respectively, when *θ *= 50 cM.

Among the different sample sizes, $N_{3Ho}^{f}$ was higher than the others and appeared as the most critical one. These dams, used for progeny testing, had to be homozygous in the QTL interval for the MS haplotype. For *θ *values below 25 cM, the number of "receiving" dams $N_{3Ho}^{f}$ was large enough to progeny test the required number of recombinant sires. When *θ *increased, the number of available locally homozygous "receiving" dams was reduced whereas the number of recombinant sires would increase. For *θ *= 25-30 cM, required $N_{3Ho}^{f}$ is equal to the number of homozygous females necessary to progeny test all the $N_{rec}^{m}$ sires. Over 30 cM, required number of progenies should be achieved by mating the "receiving" dams several times or by producing additional dams over successive generations to increase $N_{3Ho}^{f}$.

**Figure 5:**
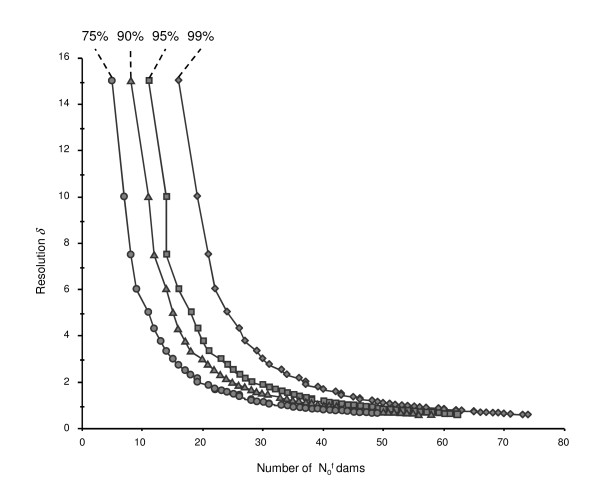
**Distribution of the mapping resolution (*δ*) depending on the number of LW dams $N_{0}^{f}$ in the case of an initial QTL interval of 30 cM, for overall probability of success of around 99%, 95%, 90% and 75%**.

Finally, computations were performed to match different risks of failure α at each step of the design, in order to achieve an overall probability of success of about 0.99, 0.95, 0.90 and 0.75. The distribution of the resolution *δ *as a function of the probability of success and the number of pure LW female used at the first generation ($N_{0}^{f}$) is shown in figure 5 in the case of an initial *θ *= 30 cM QTL interval. When the number of dams was larger than 50, a *δ *lower than 1 cM could be achieved for any probability of success considered. On the contrary, when the number of dams was low (less than 10), the resolution was always poor (higher than 6 cM), and could not be achieved with a reasonable chance of success. Large differences in resolution were observed for $N_{0}^{f}$ values between 10 and 30: using 20 LW dams led to a new resolution interval of 2 cM with a probability of success of 0.75 and 10 cM with a probability of success of 0.99. Except for a probability of success of 0.99, when the number of LW dams was higher than 30, the size of the expected new interval did not vary much, ranging from 2 to 1 cM with probabilities of success of 95% to 75%, respectively. The pattern of the distribution of *δ *was affected in the same way by the other dam sample sizes.

### Practical use of a locally congenic backcross design

Calculations carried out allowed the estimation of the number of dams necessary to produce the recombinant sires ($N_{rec}^{m}$) and the number of locally homozygous "receiving" dams ($N_{3Ho}^{f}$). In practice, when a fine mapping design using backcross families is carried out, a practical limiting factor is the number of animals, especially the number of reproductive females, that can be kept on the farm. The resolution obtained will be partly driven by the constraints of the farrowing unit. Three situations were envisaged, corresponding to capacities of 35, 50 or 100 dams. In generation G1, the whole capacity should be used in order to maximise $N_{1he}^{f}$, which determines the maximum resolution that will be achieved (Figure 4). In the following generation, the breeding capacity has to be shared between the dams $N_{2Ho}^{f}$ and $N_{2he}^{f}$. All the $N_{2Ho}^{f}$ dams should be conserved in order to produce the recombinant sires, the complementary places being used to keep $N_{2he}^{f}$ heterozygous dams. In G3, all the places can be used to keep homozygous "receiving" dams ($N_{2Ho}^{f}$ and/or $N_{3Ho}^{f}$). In the three situations considered, these dams can be used to progeny test simultaneously 3, 5 or 10 recombinant sires respectively. If the number of recombinant sires is higher than the testing capacity, the first sires have to be selected based on the position of their recombination events in the QTL interval to optimize the testing. These first families should allow the dissection of the interval in sub-intervals of similar size, resulting in the definition of a new smaller QTL interval and the selection of new recombinant sires to be progeny tested.

**Figure 6:**
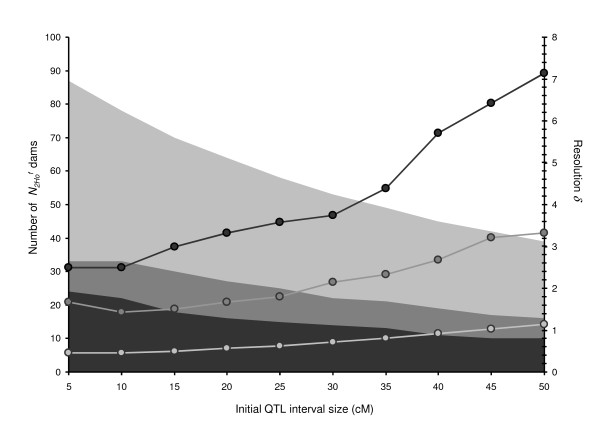
**Number of $N_{2Ho}^{f}$ dams and resolution *δ *obtained as a function of the initial interval size *θ *for three experimental farm capacities**. Scenarios corresponding to 35, 50 or 100 places for female's reproduction are respectively indicated in dark, medium and light-grey: area indicates number of homozygote MS/MS $N_{2Ho}^{f}$ dams in the G2 generation (left scale); the curves report the resolutions *δ *obtained (right scale).

In figure 6, the number of $N_{2Ho}^{f}$ dams kept and the resolution *δ *obtained were represented for different initial QTL interval sizes (*θ*) and herd capacities. The resolution obtained was quite poor with a capacity of 35 places; with an initial interval of 50 cM the expected *δ *value was 7 cM. This value was reduced to 2.5 cM if the initial interval was between 5 and 10 cM. When the number of females was equal to or above 50, the size of the confidence interval was significantly refined. The resolution *δ *varied from 3 (for *θ *= 50 cM) to around 1.5 cM (*θ *= 5 cM) with a 50 dams batch, and it was lower than 1 cM in the case of a 100 dams batch, as typical in poultry or rabbit mapping designs.

Under the scenarios studied here, the QTL genotype of a recombinant sire is evaluated based on the phenotypes of its progeny. With 100 progenies as considered here, the power to detect a QTL effect of 0.5 SD is 80%, and 90% for an effect of 0.6 SD (using a type I error rate of 5%). Hence the sample size we consider is sufficient to infer the sires QTL genotypes for QTL of moderately large effects, which is typically the kind of QTLs for which we expect this approach to be undertaken. If applied to QTL with smaller effect, the progeny size has to be increased accordingly and can be evaluated prior to performing the experiment. On the contrary, QTL with larger effect would be fine-mapped with fewer offsprings.

## Conclusions

To conclude, positioning a QTL with respect to crossovers requires knowledge of the QTL alleles carried by the recombinant sires, which is directly determined by the accuracy of QTL genotype inference from progeny testing results. We propose here the construction of an experimental design (taking into account dominance effect), using only the two F1 founder haplotypes segregating in the mapping interval (previously progeny tested in a QTL detection design). We validated this scheme with the mapping of a QTL on SSC1 chromosome. Note that such a design can be recommended even for additive QTL, by generating homozygous "testor" haplotypes in the dam herd.

Population based association studies are now performed in several species using a SNP panel of 60 thousand genome-wide markers and phenotypic data recorded in most commercial populations. It will probably accelerate the fine mapping of QTL influencing common traits, but the strategy proposed here remains a useful familial mapping strategy for QTL influencing expensive or particular phenotypes measured in experimental populations. This approach could also be adapted for QTL analysis in other species like poultry or rabbits, for which experimental crosses can easily be set up.

## Methods

### Animal and phenotypic data

Generations of backcrossing were generated at the INRA experimental farm of Le Magneraud (Surgères, Charente-Maritimes). The care and use of animals were performed in compliance with the guidelines of the French Ministry of Agriculture and Fisheries. Phenotypes of all the animals were recorded at the experimental farm and in a commercial abattoir in standard conditions. Large White sows were inseminated with semen from 3 BC2 boars (#0179-#0180-#0332) and 1 BC3 (#1357) sire issued from the INRA PorQTL program [8]. In total about 100 offspring were produced for each BC animal in order to test the segregation of QTL alleles in their progeny. The phenotypic data recorded in this study and the appropriate methods of collection have previously been described [2]. To sum up, backfat thickness using real-time ultrasound (Aloka SSD-500, Ecotron Aloka, Tokyo, Japan) was measured at 21 weeks of age, on each side of the spine at 4 cm from the middorsal line at the levels of the shoulder (neck). At approximately 105 kg, pigs were slaughtered in a commercial slaughterhouse. After slaughter, additional fat (G2) and lean (M2) depths were recorded using a Fat-o-Meat'er (SFK Technology A/S, Herlev, Denmark) probe between the 3^rd ^and 4^th ^last ribs at 6 cm off the mid-dorsal line. Meat percentage of carcass was estimated using Fat-o-Meat'er measurements.

### Markers and genotyping

Genomic DNA was purified from individual blood samples. Microsatellite markers were chosen from SSC1 consensus map based on their location in the QTL location interval and their informativity in each sire family. New markers were developed from the BAC End Sequences published for clones mapped in the QTL interval. Microsatellites MCST72H3A, MCSE153D19A, MCST104O20A, MCSE121B7A, MCST257C18A, MCSE264C2A and MCST97M13A were each developed from BES with GenBank accession number CT261471, CT239816, CT107348, CT150002, CT068826, CT234944 and CT078989, respectively. The amplifications were performed on ABI 9700 PCR machines (Applied Biosystems, Foster City, CA) and the PCR amplification analysis was carried out on an ABI 3730 automatic sequencer (Applied Biosystems). The fragment length of the PCR products and the genotype were then determined using the Genemapper software (Applied Biosystems). Results of genotyping were checked, validated, and stored in the GEMMA database [11]. A multilocus linkage map including the seven new markers, and five previously-mapped microsatellite markers was constructed using CriMap software [12] (Figure 1).

### Statistical analysis

Phenotypic data were first adjusted for environmental effects using the GLM procedure of SAS (SAS 9.1, SAS Institute, Inc.). All traits were corrected for the fixed effects of sex and batch, and ultrasonic backfat thickness was adjusted with weight at the end of the test period. QTL interval mapping was performed using QTLmap [13,14] software on the residuals of the above-mentioned model. Analyses were performed independently for each sire family assuming a half-sib model. The test statistics was computed as the ratio of likelihoods under the hypotheses of one (H1) *vs. *no (H0) QTL linked to the set of markers considered. Under the H1 hypothesis, a QTL with a gene substitution effect for each sire was fitted to the data.

### Theoretical computation

We investigated a strategy to fine map the QTL located initially in a segment S of size L centimorgans (corresponding to a recombination fraction of *θ*, using Haldane's mapping function). This strategy involved building recombinant genotypes in the segment S through the pedigree presented in figure 3.

#### Number of recombinants

We show how to estimate the population sizes required to obtain at least ($N_{rec}^{m}$) sire recombinants in generation G3. This number is determined so as to control the expected distance between recombination events in the segment. For example, if we consider ($N_{rec}^{m}$) recombinants in a segment of size L, the expected distances between crossing-overs in the region is *δ *= *θ*/($N_{rec}^{m}$ +1). We call this expected distance the resolution of the pedigree. The resulting sample sizes in each generation were determined from the number of ($N_{rec}^{m}$) sire recombinants in generation G3.

#### Calculating N4

The probability of obtaining at least ($N_{rec}^{\min}$) sire recombinants in S amongst an offspring of size N4 is:

$$P(N_{rec}^{m}\geq N_{rec}^{\min})=\sum_{k=N_{rec}^{\min}}^{N4}\left(\begin{array}{c} N4\\ k \end{array}\right){\left(\frac{1}{2}\theta \right)}^{k}{\left(1-\frac{1}{2}\theta \right)}^{N4-k}$$

Fixing a probability of failure of *α*, we can calculate the minimum size N4 that satisfies $P(N_{rec}^{m}\geq N_{rec}^{\min})>(1-\alpha )$.

#### Calculating N2

Considering that each homozygote dam at generation G2 will contribute to 10 individuals in N4, the minimum ($N_{2Ho}^{f}$) needed to obtain N4 individuals is 0.1 N4, rounded to the nearest higher integer. Given $N_{2Ho}^{f}$, we can follow the same argument to estimate N2 as we did for N4, calculating the minimum offspring size that guarantees that the probability

$$P(N_{2Ho}^{f}\geq \left\lceil 0.1N4\right\rceil )=\sum_{k=\left\lceil 0.1N4\right\rceil }^{N2}\left(\begin{array}{c} N2\\ k \end{array}\right){\left(\frac{1}{8}{\left(1-\theta \right)}^{2}\right)}^{k}{\left(1-\frac{1}{8}{\left(1-\theta \right)}^{2}\right)}^{N2-k}$$

is greater than (1-α).

#### Calculating N1

To calculate the minimum size N1, we proceed as above by noting that to obtain *N2 *individuals 0.1 $N_{1he}^{f}$ heterozygote dams must be obtained at generation G1, *i.e. *N1 is the minimum offspring size that guarantees that the probability

$$P(N_{1he}^{f}\geq \left\lceil 0.1N2\right\rceil )=\sum_{k=\left\lceil 0.1N2\right\rceil }^{N1}\left(\begin{array}{c} N1\\ k \end{array}\right){\left(\frac{1}{4}(1-\theta )\right)}^{k}{\left(1-\frac{1}{4}(1-\theta )\right)}^{N1-k}$$

is greater than (1-α).

#### Calculating N0

Finally, the number of crosses (*i.e. *the number of dams) needed at generation *G0 *is 0.1 N1, rounded to the nearest higher integer.

To evaluate the QTL genotype of the recombinant sires obtained, they will be crossed with ($N_{3Ho}^{f}$) dams. The number of such dams obtained at generation G3, can be calculated as follows:

$$\begin{array}{l} E(N_{3Ho}^{f})=10E(N_{2he}^{f})\frac{1}{4}(1-\theta )\\ E(N_{2he}^{f})=10N_{1he}^{f}\frac{1}{8}{\left(1-\theta \right)}^{2} \end{array}$$

Note that we could have constrained this number to be at least 10($N_{rec}^{m}$), but we preferred not to apply such a constraint as additional ($N_{3Ho}^{f}$) dams can be obtained with additional crossings.

In the results, we used a risk of failure *α *of 0.003, 0.015, 0.03 or 0.09 at each step, leading to an overall probability of success of about 0.99, 0.95, 0.9 and 0.75, respectively.

## Authors' contributions

JR proposed the idea, performed the synthesis of the results and drafted the manuscript. HG advised the QTL detection analysis. BS realized the calculation of the simulated design. MPS performed statistical analyses. NI genotyped the BC families. YB supervised the performance testing, from animal production to biological sampling. JPB and DM helped to draft the manuscript and had the scientific responsibility of the experiment. All authors read and approved the final manuscript.
